# Arterial and Cardiac Remodeling Associated With Extra Weight Gain in an Isolated Abdominal Obesity Cohort

**DOI:** 10.3389/fcvm.2021.771022

**Published:** 2021-11-05

**Authors:** Damien Mandry, Nicolas Girerd, Zohra Lamiral, Olivier Huttin, Laura Filippetti, Emilien Micard, Marie-Paule Bernadette Ncho Mottoh, Philip Böhme, Denis Chemla, Faïez Zannad, Patrick Rossignol, Pierre-Yves Marie

**Affiliations:** ^1^Department of Radiology, CHRU-Nancy, Université de Lorraine, Nancy, France; ^2^INSERM, UMR-1254, Université de Lorraine, Nancy, France; ^3^INSERM, UMR-1116, Université de Lorraine, Nancy, France; ^4^Department of Cardiology, CHRU-Nancy, Université de Lorraine, Nancy, France; ^5^CHRU-Nancy, INSERM, CIC 1433, Université de Lorraine, Nancy, France; ^6^Department of Endocrinology, Diabetology, Nutrition, CHRU-Nancy, Nancy, France; ^7^Explorations Fonctionnelles, Hôpital Kremlin Bicêtre, APHP, Paris, France; ^8^INSERM, UMR- 999, Hôpital Marie-Lannelongue, Le Plessis-Robinson, France; ^9^FCRIN INI-CRCT, Nancy, France; ^10^CHRU-Nancy, Université de Lorraine, Nuclear Medicine & Nancyclotep Platform, Nancy, France

**Keywords:** obesity, weight gain, arterial load, cardiac remodeling, MRI

## Abstract

**Introduction:** This study aims to assess the changes in cardiovascular remodeling attributable to bodyweight gain in a middle-aged abdominal obesity cohort. A remodeling worsening might explain the increase in cardiovascular risk associated with a dynamic of weight gain.

**Methods:** Seventy-five middle-aged subjects (56 ± 5 years, 38 women) with abdominal obesity and no known cardiovascular disease underwent MRI-based examinations at baseline and at a 6.1 ± 1.2-year follow-up to monitor cardiovascular remodeling and hemodynamic variables, most notably the effective arterial elastance (Ea). Ea is a proxy of the arterial load that must be overcome during left ventricular (LV) ejection, with increased EA resulting in concentric LV remodeling.

**Results:** Sixteen obese subjects had significant weight gain (>7%) during follow-up (WG+), whereas the 59 other individuals did not (WG–). WG+ and WG– exhibited significant differences in the baseline to follow-up evolutions of several hemodynamic parameters, notably diastolic and mean blood pressures (for mean blood pressure, WG+: +9.3 ± 10.9 mmHg vs. WG–: +1.7 ± 11.8 mmHg, p = 0.022), heart rate (WG+: +0.6 ± 9.4 min^−1^ vs. −8.9 ± 11.5 min^−1^, *p* = 0.003), LV concentric remodeling index (WG: +0.08 ± 0.16 g.mL^−1^ vs. WG−: −0.02 ± 0.13 g.mL^−1^, *p* = 0.018) and Ea (WG+: +0.20 ± 0.28 mL mmHg^−1^ vs. WG−: +0.01 ± 0.30 mL mmHg^−1^, *p* = 0.021). The evolution of the LV concentric remodeling index and Ea were also strongly correlated in the overall obese population (*p* < 0.001, R^2^ = 0.31).

**Conclusions:** A weight gain dynamic is accompanied by increases in arterial load and load-related concentric LV remodeling in an isolated abdominal obesity cohort. This remodeling could have a significant impact on cardiovascular risk.

## Introduction

Obese subjects exhibit an unequivocal increase in cardiovascular risk (1). Unfortunately, one-half of these subjects are not even attempting to lose weight, and many succumb to even further weight gains (2). Such weight gains lead to body mass indexes associated with higher cardiovascular risks (1), with the prognosis being further deteriorated by a longitudinal weight gain dynamic, independently of the attained bodyweight level (3–5).

A weight gain dynamic was also found to be associated with the onset or exacerbation of a phenotype involving concentric left ventricular (LV) remodeling, with an increase in LV mass relative to the LV cavity size, in large cohorts of obese as well as non-obese subjects (6, 7). The specific prognostic impact of this concentric remodeling, as well as its associations with concomitant obesity-related arterial remodeling and increasing blood pressure (BP), need to be specifically determined in obese subjects. In more general populations, concentric LV remodeling was previously shown to be a strong prognostic indicator (8–12), developing in response to increases in myocardial wall stress (13) and arterial load (i.e., the artery-related opposition that must be overcome by the LV during ejection) (14).

Magnetic resonance imaging (MRI) measures cardiac and vascular function in a more precise and reproducible way than echography (15). This may involve measurements of LV mass and cavity volumes, as well as compliance, elastance, and resistance of the systemic arterial tree (14–17). In previous cross-sectional MRI studies, middle-aged subjects with isolated abdominal obesity exhibited significant deterioration in large vessel compliance and an increase in the vascular resistance of small resistive vessels, as compared with non-obese subjects, and this was accompanied by an LV concentric remodeling (17). However, longitudinal studies assessing the interrelated cardiac and vascular changes attributable to the additional weight gain over time are still lacking in obese subjects.

This MRI-based longitudinal study assesses the cardiovascular changes and remodeling attributable to significant additional weight gain over time in a middle-aged isolated abdominal obesity cohort.

## Materials and Methods

### Study Populations

As already detailed elsewhere (17), isolated abdominal obesity subjects were prospectively recruited through an advertising campaign and ultimately included subjects ranging from 40 to 65 years of age, with obvious abdominal obesity defined by a waist circumference >102 cm for men and >88 cm for women (18), and excluding any of the following: (1) morbid obesity [i.e., body mass index (BMI) > 40 Kg.m^−2^], (2) a history of any cardiovascular disease or of any medical treatment with cardiovascular effects, (3) a history of medically-treated hypertension or diabetes, (4) inflammatory disease, (5) renal, hepatic or pulmonary insufficiency, (6) an MRI contraindication, (7) absence of cardiac sinus rhythm and (8) any women of childbearing potential. An additional control group of non-obese healthy volunteers with a similar sex ratio and age range (40–65 years), had also been prospectively recruited through an unrelated concurrent advertising campaign (17). These included waist circumferences < 94 cm for men and < 80 cm for women in order to exclude any central obesity cases (17).

The transversal part of this exploratory study, involving a cardiovascular MRI investigation, was approved by the local Ethics Committee, with all study participants providing their signed informed consent. We have previously published the baseline results for the isolated abdominal obesity cohort elsewhere (17).

The same obese subjects were subsequently asked to participate in an additional >4 years longitudinal study, which included a follow-up using the same cardiovascular MRI protocol applied in the baseline study. The Ethics Committee also approved this longitudinal part of the study, which is released on the ClinicalTrials.gov site under the identifier NCT01716819. All study subjects additionally gave their signed informed consent to participate in this longitudinal part of the study.

Significant weight gain during follow-up was defined by the standard criterion of a > 7% increase relative to baseline (19–22).

### Cardiovascular MRI

As previously detailed elsewhere (17), MRIs were performed on a 1.5-T magnet (Signa Excite, GE Medical Systems, Milwaukee, WI, USA). During MRI examinations, an automated sphygmomanometer (Maglife C, Schiller Medical, Wissembourg, France) was used to measure brachial blood pressure (BP) as systolic, diastolic, and mean pressures. Averaged values were archived and extracted at a later date to perform analyses presented below.

A conventional steady-state free precession pulse sequence was used to assess LV function and mass in contiguous short-axis 8-mm slices, with 30 phases per cardiac cycle, a 32 to 38-cm field-of-view, and a 224x224 matrix (17). LV end-diastolic volume, end-diastolic mass, and ejection fraction were obtained using dedicated software (MASS™, Medis, The Netherlands), with papillary muscles and trabeculations being excluded from LV mass. The concentric remodeling (CR) index was defined as LV mass/end-diastolic volume ratio (17). The intra-observer reproducibility of these remodeling LV parameters had been previously assessed in 31 Cardiovascular Magnetic Resonance (CMR) exams that were analyzed twice. Absolute values of the differences between the first and second measurements were on average 5.81 ± 4.17 g for LV mass, 4.68 ± 3.64 ml for end-diastolic volume, and 0.060 ± 0.046 for the CR index.

The cardiac flow was determined using a conventional velocity-encoded phase-contrast gradient-echo sequence on a single 10-mm slice positioned perpendicularly to the ascending aorta, 32 phases per cardiac cycle, and a unidirectional velocity with a maximum set to 1.50 m sec^−1^ (17). The stroke volume (SV) was obtained using the “CV flow” software (Medis, The Netherlands) and automatic contour detection. Velocities were only corrected using an ROI-based method in instances of obvious offset error.

The values of cardiac flow and stroke volume were used to estimate the systemic vascular resistances (SVR: mean pressure/cardiac flow) (14–17), as well as two additional vascular parameters: (1) effective arterial elastance (Ea), a proxy for the arterial load, which needs to be overcome during left ventricular (LV) ejection (Ea = 0.9 x systolic BP (mmHg) / stroke volume (mL) (23–28), and (2) the total arterial compliance index (TAC = stroke volume (mmHg) / pulse pressure (mmHg) (14, 16, 17, 27, 28). These cardiovascular MRI-derived parameters were not indexed to anthropometric parameters except for transversal unpaired comparisons between the obese and non-obese groups (Table 1) where several variables were indexed to body surface area and LV mass to body weight to the 2.7 power [weight^2.7^ (29)].

**Table 1:** Comparison of the main baseline data between obese subjects and non-obese healthy controls.

	**Obese**	**Non-obese**	***P* value**
	**(*n* = 75)**	**(*n* = 58)**	
Age (years)	55 ± 6	54 ± 5	0.245
Female gender	38 (51%)	31 (53%)	0.861
Body weight (kg)	89.1 ± 11.8	63.8 ± 8.3	<0.001
Body mass index (kg m^−2^)	31.7 ± 3.3	22.4 ± 1.9	<0.001
Heart rate (bpm)	72 ± 11	69 ± 11	0.081
Systolic BP (mmHg)	121.4 ± 14.7	118.6 ± 15.2	0.283
Diastolic BP (mmHg)	72.0 ± 10.3	76.9 ± 9.3	0.005
Mean BP (mmHg)	88.5 ± 10.8	90.8 ± 11.1	0.222
Pulse BP (mmHg)	49.4 ± 10.5	41.7 ± 7.3	<0.001
Indexed stroke volume (mL m^−2^)	41.5 ± 6.8	47.4 ± 8.5	<0.001
Cardiac index (L.min^−1^ m^−2^)	2.90 ± 0.61	3.23 ± 0.69	0.004
Indexed SVR (mmHg min m^2^ L^−1^)	31.7 ± 7.3	29.1 ± 5.8	0.024
Indexed TAC (mL mmHg^−1^ m^−2^)	0.85 ± 0.19	1.16 ± 0.25	<0.001
Indexed Ea (mmHg mL^−1^ m^2^)	2.50 ± 0.54	2.08 ± 0.43	0.001
Indexed EDV (mL m^−2^)	69.8 ± 10.7	81.2 ± 10.0	<0.001
EF (%)	59.5 ± 6.1	59.9 ± 5.8	0.692
Indexed LV mass (g m^−2.7^)	24.0 ± 4.7	21.8 ± 3.8	<0.001
CR index (g mL^−1^)	0.69 ± 0.15	0.63 ± 0.12	0.005

*BP, blood pressure; CR, concentric remodeling; Ea, effective arterial elastance; EDV, end-diastolic volume; EF, ejection fraction; LV, left ventricle; TAC, total arterial compliance; SVR, systemic vascular resistances*.

*Note that all MRI parameters are indexed to body surface area here, except for EF and CR index*.

### Statistical Analysis

Analyses were performed using the commercially available SAS software, version 9.4 (SAS Institute Inc. Cary, NC, USA). Continuous variables were expressed as means (± standard deviation) and categorical variables as numbers and percentages. Unpaired comparisons for categorical variables were performed with Fisher's exact test, and unpaired comparisons of continuous variables were evaluated with Student's *t*-test or the Mann–Whitney U test, depending on their normal or non-normal distributions. Comparisons between baseline and follow-up within each group (WG+, WG–) were also evaluated using the signed rank test or the paired *t*-test for continuous variables and the Mc Nemar test for categorical variables. Linear regression analyses were additionally carried out to investigate and assess the relationships between certain variables. Additional predictions provided by the blood pressure data were analyzed using an ascending stepwise multivariate regression model. Linear model assumptions were checked, and *p* < 0.05 were considered to indicate a significant difference.

## Results

### Baseline Characteristics of Obese Subjects Compared to Non-obese Subjects

Seventy-five subjects with abdominal obesity (56 ± 5 years, 38 women), who underwent the MRI protocol at baseline and follow-up, were ultimately considered in the analysis. Of this group, 50 (67%) also had general obesity, as defined by a body mass index > 30 kg m^−2^. As detailed in Table 2, eight individuals (11%) were taking hypolipidemic medication, and none were on anti-hypertensive treatments.

**Table 2:** Main data collected at baseline and follow-up in the overall obese population, as well as in obese subjects exhibiting significant weight gain (WG+) or not (WG-), with *p* values for paired comparisons between baseline and follow-up.

	**Overall obese population (*****n*** **= 75)**			**WG+ (*****n*** **= 16)**			**WG– (*****n*** **= 59)**		
	**Baseline**	**Follow-up**	***P* value**	**Baseline**	**Follow-up**	***P* value**	**Baseline**	**Follow-up**	***P* value**
Body weight (kg)	89 ± 12	91 ± 14	0.034	87 ± 14	99 ± 15*	<0.0001	90 ± 11	89 ± 13	0.64
Antihypertensive drugs	0 (0.0 %)	21 (28.0 %)	<0.0001	0 (0.0 %)	4 (25.0 %)	0.12	0 (0.0 %)	17 (28.8 %)	<0.0001
Beta-blockers	0 (0.0 %)	8 (10.7 %)	0.0078	0 (0.0 %)	2 (12.5 %)	0.50	0 (0.0 %)	6 (10.2 %)	0.0313
ACEI or ARA-II drugs	0 (0.0 %)	14 (18.7 %)	0.0001	0 (0.0 %)	3 (18.8 %)	0.25	0 (0.0 %)	11 (18.6 %)	0.001
Antidiabetic drugs	0 (0.0 %)	2 (2.7 %)	0.50	0 (0.0 %)	0 (0.0 %)	——	0 (0.0 %)	2 (3.4 %)	0.50
Hypolipidemic drugs	8 (10.7 %)	16 (21.3 %)	0.0215	6 (37.5 %)**	7 (43.8 %)*	1.00	2 (3.4 %)	9 (15.3 %)	0.0156
Statins	6 (8.0 %)	12 (16.0 %)	0.0339	4 (25.0 %)*	7 (43.8 %)**	0.0833	2 (3.4 %)	5 (8.5 %)	0.18
CRP (g L^−1^)	3.25 ± 3.03	4.26 ± 6.21	0.17	3.29 ± 2.49	4.62 ± 4.57	0.21	3.24 ± 3.20	4.16 ± 6.62	0.37
Blood creatinine (mmol L^−1^)	84.0 ± 12.4	84.9 ± 17.5	0.63	85.4 ± 11.5	84.3 ± 15.8	0.73	83.6 ± 12.7	85.1 ± 18.0	0.43
HbA1c (%)	5.63 ± 0.33	5.78 ± 0.57	0.0002	5.66 ± 0.38	5.85 ± 0.42	0.023	5.62 ± 0.31	5.76 ± 0.61	0.005
LDL (mmol L^−1^)	3.58 ± 0.91	3.34 ± 0.80	0.27	3.36 ± 0.58	3.20 ± 0.64	0.43	3.65 ± 0.98	3.38 ± 0.84	0.43
HDL (mmol L^−1^)	1.41 ± 0.36	1.26 ± 0.30	<0.0001	1.48 ± 0.41	1.24 ± 0.28	0.0006	1.39 ± 0.34	1.27 ± 0.31	<0.0001
Triglycerides (mmol L^−1^)	1.56 ± 0.92	1.67 ± 1.14	0.18	1.59 ± 1.03	2.02 ± 1.15	0.009	1.55 ± 0.89	1.57 ± 1.12	0.97
Heart rate (bpm)	72 ± 11	65 ± 12	<0.0001	71 ± 11	72 ± 12**	0.82	72 ± 11	63 ± 11	<0.0001
Systolic BP (mmHg)	121 ± 15	124 ± 13	0.018	119 ± 12	128 ± 12	0.009	122 ± 15	123 ± 13	0.18
Diastolic BP (mmHg)	72 ± 10	76 ± 10	0.0001	69 ± 13	80 ± 11	0.006	73 ± 10	75 ± 10	0.005
Mean BP (mmHg)	88 ± 11	92 ± 10	0.001	86 ± 12	95 ± 11	0.009	89 ± 11	91 ± 9	0.025
Pulse BP (mmHg)	49 ± 10	48 ± 9	0.16	50 ± 8	48 ± 10	0.5	49 ± 11	48 ± 9	0.28
Stroke volume (mL)	82 ± 15	83 ± 19	0.78	84 ± 12	79 ± 18	0.21	82 ± 16	84 ± 19	0.37
Cardiac output (L min^−1^)	5.88 ± 1.24	5.26 ± 1.10	<0.0001	5.93 ± 1.07	5.59 ± 1.29	0.19	5.87 ± 1.30	5.17 ± 1.04	<0.0001
SVR (mmHg min L^−1^)	15.7 ± 3.7	18.1 ± 3.7	<0.0001	15.0 ± 3.4	17.8 ± 4.0	0.003	15.9 ± 3.7	18.2 ± 3.6	<0.0001
TAC index (mL mmHg^−1^)	1.72 ± 0.42	1.78 ± 0.49	0.25	1.70 ± 0.30	1.70 ± 0.43	0.94	1.73 ± 0.45	1.80 ± 0.51	0.22
Ea (mmHg mL^−1^)	1.23 ± 0.27	1.28 ± 0.33	0.16	1.18 ± 0.22	1.38 ± 0.35	0.013	1.25 ± 0.28	1.26 ± 0.33	0.81
EDV (mL)	142 ± 26	140 ± 32	0.16	144 ± 21	138 ± 29	0.27	141 ± 27	140 ± 32	0.32
EF (%)	60 ± 6	59 ± 5	0.29	60 ± 7	58 ± 7	0.13	59 ± 6	59 ± 5	0.64
LV mass (g)	98 ± 25	96 ± 26	0.061	97 ± 28	103 ± 30	0.43	98 ± 25	94 ± 24	0.013
CR index (g.mL^−1^)	0.69 ± 0.15	0.70 ± 0.14	0.89	0.67 ± 0.16	0.75 ± 0.18	0.13	0.70 ± 0.14	0.68 ± 0.13	0.25

Significant results for unpaired comparisons between WG+ and WG− at baseline or follow-up are also indicated in the WG+ columns (*p <0.05 and **p <0.005 for comparison with WG−).

*BP, blood pressure; CR, concentric remodeling; CRP, c reactive protein; Ea, effective arterial elastance; EDV, end-diastolic volume; EF, ejection fraction; HbA1c, hemoglobin A1c; HDL, high density lipoprotein; LDL, low density lipoprotein; LV, left ventricular; TAC, total arterial compliance; SVR, systemic vascular resistances*.

Although obese subjects had a similar age range and sex ratio than the non-obese group at baseline, the obese group exhibited higher diastolic and pulse BP, with a trend toward higher heart rates (Table 1). The obese group also showed signs of LV concentric hypertrophy (i.e., higher CR indexes and indexed LV mass compared to the non-obese controls), and arterial dysfunction with lower arterial compliance and higher levels of vascular resistance as well as arterial load [i.e., with indexed TAC, SVR and Ea values being significantly different from the corresponding values in non-obese controls (Table 1)].

### Six-Year Follow-Up of Obese Subjects

The follow-up investigation was performed at a mean of 6.1 ± 1.2 years from baseline. Several variables significantly deteriorated over time in the overall obese population, notably body weight, BP, SVR, HbA1c and HDL cholesterol (Table 2). However, only 16 obese subjects experienced significant weight gain, as defined by the standard criterion of a > 7% increase relative to baseline (19–22) (WG+ group: from 87 ± 14 kg to 99 ± 15 kg, *p* < 0.001), whereas the 59 other individuals had no significant bodyweight changes (WG– group: from 90 ± 11 kg to 89 ± 13 kg, *p* = 0.64).

The WG+ and WG– subgroups were comparable with respect to age (at baseline: 54.0 ± 6.2 vs. 55.3 ± 5.5 years, *p* = 0.41), gender (women: 9 (56%) vs. 29 (49%), *p* = 0.78), baseline body mass index (31.5 ± 3.5 vs. 31.8 ± 3.3 kg.m^−2^, *p* = 0.75) and time between baseline and follow-up (6.1 ± 1.2 vs. 6.1 ± 1.2 years, *p* = 0.98). However, as detailed in Table 2, the WG+ group exhibited higher rates of hypolipidemic treatments, particularly of statins compared to the WG- group (for statins at follow-up: 7 (43.8 %) vs. 5 (8.5 %), *p* = 0.0024).

**Figure 1:**
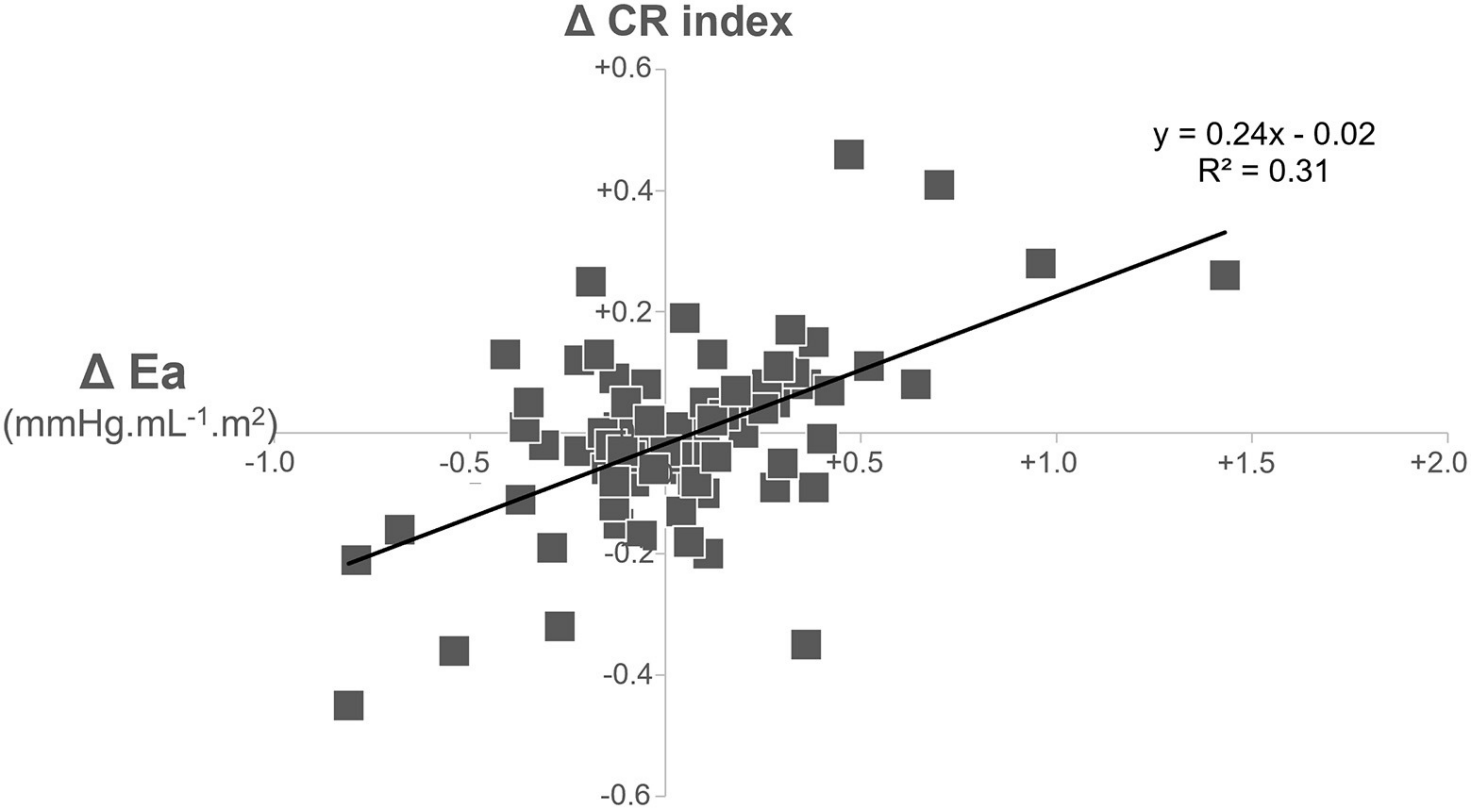
Correlation between baseline to follow-up changes in effective arterial elastance (Ea) and in the left ventricular concentric remodeling (CR) index in the overall population of obese subjects.

These two groups also exhibited significant differences in the baseline to follow-up evolutions of several hemodynamic parameters (Tables 2, 3), notably for mean and diastolic BP (for mean BP, WG+: +9.3 ± 10.9 mmHg vs. WG-: 1.7 ± 11.8 mmHg, p = 0.022), heart rate (WG+: +0.6 ± 9.4 min^−1^ vs. −8.9 ± 11.5 min^−1^, *p* = 0.003), LV concentric remodeling index (WG: +0.080 ± 0.16 g mL^−1^ vs. WG-: −0.02 ± 0.13 g mL^−1^, *p* = 0.018) and Ea (WG+: +0.20 ± 0.28 mL.mmHg^−1^ vs. WG-: +0.01 ± 0.30 mL mmHg^−1^, *p* = 0.021). In addition, the evolutions of the LV concentric remodeling index and the Ea were found to be strongly correlated in the overall obese population [*p* < 0.001, R^2^ = 0.31 (Figure 1)]. In contrast, evolution of the LV concentric remodeling index was unrelated to the concomitant changes in brachial blood pressure in the univariate, as well as the multivariate analysis (-i.e., after Ea was entered in a linear regression model).

**Table 3:** Comparison between the obese subjects exhibiting significant weight gain (WG+) and those without weight gain (WG–), of the parameter differences calculated between follow-up and baseline.

	**WG+ (*n* = 16)**	**WG– (*n* = 59)**	***P* value**
Body weight (kg)	11.73 ± 7.55	−0.98 ± 5.45	<0.0001
CRP (g L^−1^)	1.32 ± 4.16	0.27 ± 2.92	0.26
Blood creatinine (mmol L^−1^)	−1.1 ± 11.4	1.8 ± 10.3	0.33
HbA1c (%)	0.19 ± 0.32	0.18 ± 0.51	0.28
LDL (mmol L^−1^)	−0.16 ± 0.71	−0.23 ± 1.02	0.73
HDL (mmol L^−1^)	−0.24 ± 0.22	−0.12 ± 0.19	0.086
Triglycerides (mmol L^−1^)	0.42 ± 0.66	0.05 ± 0.69	0.058
Heart rate (bpm)	0.6 ± 9.4	−8.9 ± 11.5	0.003
Systolic BP (mmHg)	8.4 ± 11.2	1.4 ± 15.4	0.097
Diastolic BP (mmHg)	10.6 ± 12.0	2.8 ± 11.0	0.027
Mean BP (mmHg)	9.3 ± 10.9	1.7 ± 11.8	0.022
Pulse BP (mmHg)	−2.2 ± 6.7	−1.4 ± 10.0	0.95
Stroke volume (mL)	−4.8 ± 14.2	1.6 ± 15.6	0.15
Cardiac output (L min^−1^)	−0.3 ± 1.0	−0.7 ± 1.0	0.19
SVR (mmHg min L^−1^)	2.86 ± 3.57	2.27 ± 3.49	0.66
TAC (mL mmHg^−1^)	−0.00 ± 0.28	0.07 ± 0.43	0.52
Ea (mmHg mL^−1^)	0.20 ± 0.28	0.01 ± 0.30	0.021
EDV (mL)	−5.14 ± 22.59	−1.40 ± 21.10	0.537
EF (%)	−1.73 ± 4.47	−0.24 ± 5.49	0.27
LV mass (g)	6.24 ± 18.91	−3.60 ± 13.56	0.1
CR index (g mL^−1^)	0.08 ± 0.16	−0.02 ± 0.13	0.018

*BP, blood pressure; CR, concentric remodeling; CRP, c reactive protein; Ea, effective arterial elastance; EDV, end-diastolic volume; EF, ejection fraction; HbA1c, hemoglobin A1c; HDL, high density lipoprotein; LDL, low density lipoprotein; LV, left ventricular; TAC, total arterial compliance; SVR, systemic vascular resistances*.

Finally, no other differences were observed between the WG+ and WG– groups over the 6-year follow-up period in terms of the evolution of any of the other clinical, biological and MRI parameters monitored (Tables 2, 3).

## Discussion

The increased cardiovascular risk of obese subjects has been well established, in line with various metabolic disorders and in association with cardiac remodeling, which has been extensively analyzed in previous transversal studies. However, to the best of our knowledge, the current MRI study is the first to assess cardiac remodeling in obese subjects along with its dependence on concomitant vascular function and with or without significant weight gain over time. Such weight gain was associated with additional increases in arterial load and load-related cardiac remodeling, and these changes are indicative of a real impact on cardiovascular function and on cardiovascular risk.

The current study analyzed arterial remodeling by measuring MRI-based parameters, more specifically: (i) the compliance attributable to great arteries [TAC index (14, 16, 17, 27, 28)], (ii) the resistance attributable to microcirculation [SVR (14–17), and (iii) a global arterial load, which is impacted by both arterial compliance and arterial resistance [Ea (23–28)]. All these parameters were markedly different between our subjects with isolated abdominal obesity and a non-obese reference population at baseline (Table 1).

Moreover, SVR and BP exhibited additional deteriorations after a mean 6-year follow-up in our obese subjects (Table 2), confirming the central function of the microvasculature in obesity-related vascular remodeling (30). Over time, these deteriorations were associated with increased use of hypertensive and hypolipidemic medications and a worsening of several other metabolic parameters (namely, HbA1c and HDL cholesterol) (see Table 2). Although the aging process may partially explain these deteriorations, they were likely accelerated by obesity in this instance.

The aggravation of obesity, characterized by significant weight gain over a 6-year time frame, affected 21% of our obese population, and its only significant predictive factor was a higher prescription rate of hypolipidemic drugs, particularly of statins (Table 2). Statins have previously been reported to be associated with weight gain, but a mechanism to substantiate this effect is still a matter of much debate (31).

We also noted time-related increases in Ea and in the LV concentric remodeling index in our obese subjects with weight gain but not in those without weight gain. Ea is a steady-state arterial parameter that provides a comprehensive measure of the vascular load impacting the LV contraction (23–28). That is why this parameter is commonly associated with an LV concentric remodeling in both transversal and longitudinal studies in men (14) as well as in animal models (32). In our study this consideration is strengthened by the strong correlation observed between the 6-year changes in Ea and the LV concentric remodeling index (see Figure 1).

This LV concentric remodeling index is an established independent prognostic parameter (8–12). It reflects the LV adaptation to an excessive LV afterload, such as in hypertensive or pre-hypertensive states (14, 33) and in patients with aortic stenoses, and contributes to prevent excess systolic LV wall stress (34). A high LV concentric remodeling index also reflects an inappropriate LV mass relative to the LV cavitary volume. In support of this consideration, an index of the appropriateness of LV mass was additionally computed relative to the normal reference values using an echography method (35–37). In the present study, this appropriateness index provided equivalent results than our concentric remodeling index - i.e., higher values in obese than in non-obese subjects, and follow-up increases correlated with weight gain in obese subjects (results not shown).

A sympathetic overactivity has also been previously found to occur during time periods characterized by weight gain in animal models (38) and in non-obese subjects (39). This may potentially play a role in the weight gain-related hemodynamic alterations documented in our study, including changes in heart rate.

Indeed, the aging-related decrease in heart rate, which is usually documented in the decade ranging from 50 to 60 years of age (6, 40), was clearly observed in our obese subjects without weight gain but not in those with weight gain. This observation strengthens the hypothesis of a difference in the activity of the sympathetic nervous system between these two groups. However, future studies which include more direct measurements of this sympathetic activity will be required to confirm the nature of this correlation. If this were to be confirmed, drugs which lower sympathetic activation could be tested to lower this weight gain-related increase in arterial load and concentric LV remodeling. Beta-blockers with additional vasodilating properties and with neutral or beneficial effects on insulin sensitivity and lipid metabolism (41), may be particularly interesting for this purpose.

The current study has several limitations, the first being its small sample size and, consequently, the necessity to confirm the results in a larger cohort. A second limitation is that cardiovascular remodeling may be impacted by variables that could only be poorly or not at all taken into account here -i.e., changes in comorbidities, drug therapies and physical activity throughout the long follow-up period; and the impact of aging compared to that observed in a non-obese population of the same age range. Another limitation is the uncertainty regarding the actual clinical impact of the cardiovascular changes specifically related to additional weight gain. Indeed, several vascular and blood metabolic parameters have been observed to deteriorate over time in the absence of weight gain (Table 2), and these parameters may at least have an equivalent prognostic importance than the weight gain-related parameters (i.e., concentric remodeling index and effective arterial elastance).

CMR is not a widely available worldwide, and although our study measured LV concentric remodeling and Ea by CMR, these parameters may also be assessed using echography-Doppler techniques (25, 26). However the accuracy of these measurements is likely to be lower for echography-Doppler compared to CMR. Echography-Doppler nevertheless has the advantage of an easier evaluation of the LV diastolic function, an additional prognostic indicator (41), which is frequently affected in obese subjects (42). Unfortunately, the LV diastolic function could not be assessed in the present CMR study, which constitutes an additional limitation.

A final limitation is the absence of quantification of epicardial adipose tissue, a parameter that might significantly impact cardiovascular hemodynamics (43).

## Conclusion

This MRI study shows that time periods characterized by significant weight gain are associated with additional increases in arterial load and load-related concentric LV remodeling in subjects with isolated abdominal obesity. Such changes indicate a real increase in cardiovascular risk, particularly for concentric LV remodeling, a recognized strong prognostic indicator (8–12).

## Data Availability Statement

The raw data supporting the conclusions of this article will be made available by the authors, without undue reservation.

## Ethics Statement

The studies involving human participants were reviewed and approved by Comité de Protection des Personnes Nancy Est. The patients/participants provided their written informed consent to participate in this study.

## Author Contributions

DM, NG, ZL, and P-YM contributed significantly to the analysis and interpretation of the data. DM, DC, FZ, PR, and P-YM contributed to the writing of the manuscript and revision of the manuscript. OH, LF, EM, MN, and PB collaborated in the study implementation, and/or management of the included subjects. All authors contributed to the article and approved the submitted version.

## Funding

This study was funded by a National Health Ministry (Programme Hospitalier de Recherche Clinique) and the 6th framework program of the European Commission (Ingenious HyperCare Network of Excellence; contract number LSHM-CT-2006-037093).

## Conflict of Interest

NG, ZL, EM, FZ, and PR were employed by company CHRU-Nancy, INSERM, CIC. The remaining authors declare that the research was conducted in the absence of any commercial or financial relationships that could be construed as a potential conflict of interest.

## Publisher's Note

All claims expressed in this article are solely those of the authors and do not necessarily represent those of their affiliated organizations, or those of the publisher, the editors and the reviewers. Any product that may be evaluated in this article, or claim that may be made by its manufacturer, is not guaranteed or endorsed by the publisher.
